# Laboratory testing for cytomegalovirus among pregnant women in the United States: a retrospective study using administrative claims data

**DOI:** 10.1186/1471-2334-12-334

**Published:** 2012-12-03

**Authors:** Jessica Leung, Michael J Cannon, Scott D Grosse, Stephanie R Bialek

**Affiliations:** 1 National Center for Immunization and Respiratory Diseases, Centers for Disease Control and Prevention, 1600 Clifton Rd NE, Atlanta, GA 30333, USA; 2 National Center for Birth Defects and Developmental Disabilities, Centers for Disease Control and Prevention, 1600 Clifton Rd NE, Atlanta, GA 30333, USA

**Keywords:** CMV, Cytomegalovirus, MarketScan, Pregnant women, Screening, Laboratory testing

## Abstract

**Background:**

Routine cytomegalovirus (CMV) screening during pregnancy is not recommended in the United States and the extent to which it is performed is unknown. Using a medical claims database, we computed rates of CMV-specific testing among pregnant women.

**Methods:**

We used medical claims from the 2009 Truven Health MarketScan® Commercial databases. We computed CMV-specific testing rates using CPT codes.

**Results:**

We identified 77,773 pregnant women, of whom 1,668 (2%) had a claim for CMV-specific testing. CMV-specific testing was significantly associated with older age, Northeast or urban residence, and a diagnostic code for mononucleosis. We identified 44 women with a diagnostic code for mononucleosis, of whom 14% had CMV-specific testing.

**Conclusions:**

Few pregnant women had CMV-specific testing, suggesting that screening for CMV infection during pregnancy is not commonly performed. In the absence of national surveillance for CMV infections during pregnancy, healthcare claims are a potential source for monitoring practices of CMV-specific testing.

## Background

Cytomegalovirus (CMV) is the most common congenital viral infection in the United States and a leading cause of congenital hearing loss and neurological disability [1]. CMV can be transmitted to the fetus when a CMV seronegative woman develops a primary CMV infection during pregnancy, or from latent virus reactivation from maternal CMV infection acquired prior to pregnancy or re-infection with a new CMV strain during pregnancy. The risk of CMV transmission to the fetus is higher among pregnant women with primary infection compared to those who were IgG positive prior to pregnancy, IgG positive at their first pregnancy visit, or IgM positive with high IgG avidity and therefore presumed to have non-primary infection (30-40% compared to 0.2-2%) [2]. Although vertical transmission is more likely to occur as a result of maternal infections in the third trimester, the rate of permanent sequelae from CMV is lower among infants infected during the third trimester than among infants born to mothers with primary CMV infection in the first trimester [3,4]. In the United States, approximately 42-50% of women 20–49 years of age are CMV seronegative [5], and it is estimated that 27,000 primary CMV infections occur among pregnant women each year [6].

Most CMV infections in immunocompetent persons are asymptomatic or present with non-specific symptoms [7,8], with only a minority of persons experiencing mononucleosis [8]. Diagnosis of CMV infection among otherwise healthy adults generally relies on serologic testing; proposed algorithms have included documentation of seroconversion or detection of CMV-specific IgM antibody in association with low IgG avidity [4]. However confirmation of CMV seroconversion during pregnancy has practical challenges [9]. Demonstrating seroconversion requires multiple blood draws over time as CMV IgM testing alone is not adequately specific for diagnosing primary infection. It does not allow for reliable determination of the timing of infection, and in the case of a pregnant woman, does not provide information on whether CMV infection occurred before or after the start of pregnancy. IgG avidity may provide information on a window of time during which primary infection may have occurred; however, most commercial laboratories in the United States do not currently offer CMV IgG avidity testing [10]. When primary maternal CMV infection is diagnosed or suspected, additional diagnostic testing, including PCR or viral culture of either amniotic fluid or fetal blood and fetal ultrasound or magnetic resonance imaging, may allow for determination of whether fetal infection has occurred.

There is currently no recommendation for routine CMV screening during pregnancy by any professional association or national public health authority [11,12] and provider and public awareness of congenital CMV infection is low [13-16]. An uncontrolled study suggested that CMV hyperimmune globulin administered during pregnancy may help reduce the risk of congenital CMV infection [17] and additional data from controlled clinical studies may inform future treatment options [18-20]. The extent to which prenatal screening or diagnostic testing for CMV is occurring in the United States is unknown and there is currently little information on national practices around CMV testing during pregnancy. Identifying testing practices will provide useful information to monitor future screening and prevention programs. We used a large healthcare claims database to explore current practices and rates of CMV testing among pregnant women in the United States.

## Methods

### Data source

The Truven Health MarketScan® Commercial Databases (Truven Health MarketScan Databases, Truven Health Analytics, Ann Arbor, MI) are derived from insurance claims for almost 40 million employees and their beneficiaries in the United States [21]. We used outpatient and inpatient claims data from the 2009 MarketScan Commercial Claims and Encounters databases, including information on demographics, health plan membership, International Classification of Diseases-9^th^ revision, Clinical Modification (ICD-9-CM) codes, and Current Procedural Terminology (CPT) codes. This study was reviewed by the human subjects research coordinator at the CDC and, as an analysis of secondary data without personal identifiers, was determined not to require institutional review board review.

### Study definitions

We defined pregnant women as women aged 15–44 years enrolled for 365 days in 2009 with their first delivery code Appendix identified during October-December 2009 in order to ensure that enrollees’ medical claims for the entire pregnancy were captured. The delivery date was approximated using the date of the first claim with a delivery code. We assumed that pregnancies lasted for no longer than 42 weeks and defined pregnancy-associated claims as those occurring in the 42 weeks before the first claim with a delivery code. We classified claims which occurred during 0–13 weeks to be within the 1^st^ trimester, those within weeks 14–26 to be in the 2^nd^ trimester, and those within weeks 27–42 to be in the 3^rd^ trimester. A prenatal visit was defined as a medical claim with a code consistent with prenatal care Appendix. Prenatal care was defined as ≥2 prenatal visits in the 1^st^ trimester or ≥1 global billing codes for prenatal care (CPT 59400, 59425, 59426, 59510, 59610) anytime during the pregnancy. We defined CMV-specific testing as a claim for CMV IgG, IgM, direct fluorescent testing (DFA), enzyme immunoassay (EIA), or polymerase chain reaction (PCR) Appendix. Potential cases of CMV infection or disease were defined as pregnant women with an ICD-9-CM code for CMV disease (078.5); cases of infectious mononucleosis were defined as pregnant women with an ICD-9-CM code for mononucleosis (075). Since mononucleosis is a potential symptom and possible indication of maternal CMV infection [8], we examined rates of pregnant women with a diagnostic code for mononucleosis. Although infectious mononucleosis is a clinical syndrome commonly associated with primary Epstein-Barr virus infection during or after second decade of life [22], adults with primary CMV infection may also develop mononucleosis-like syndromes [23]. Evidence of laboratory confirmation of CMV infection was not included in the CMV case definition because laboratory testing results were not available in the MarketScan database. We defined urban residence based on an enrollee’s Metropolitan Statistical Area (MSA) status.

### Statistical analysis

We computed frequencies of pregnant women with a code for CMV disease and CMV-specific testing to evaluate whether a pregnant woman had a diagnostic code for CMV disease based on clinical assessment alone, or also in combination with laboratory testing. We performed Pearson Chi Square or Fisher’s exact test to examine whether there was an association between CMV-specific testing and age, region, residence in an urban area, and diagnosis of infectious mononucleosis. Variables with a p-value <0.05 were considered significant. The data were analyzed using SAS (version 9.2; SAS Institute Inc, Cary, NC).

To better understand the possible sensitivity of the 2009 MarketScan Commercial databases for detecting claims for CMV-specific laboratory testing, we calculated frequencies of pregnant women with ≥1 codes for other laboratory tests that are recommended by the American College of Obstetricians and Gynecologists to be routinely performed during pregnancy [24]. These sub-groups included pregnant women who had claims for rubella, anemia, urine, glucose, syphilis, hepatitis B, chlamydia, and HIV testing Appendix. Among these women, we also calculated the frequency of pregnant women with a code for CMV-specific testing.

## Results

There were 77,773 deliveries that occurred among MarketScan Commercial enrollees during October-December 2009; 92% of these deliveries occurred in women aged 20–39 years. Characteristics of these women are shown in Table 1. Among these, 54,925 (71%) pregnant women received prenatal care (≥2 prenatal-coded claims in the 1^st^ trimester or global billing code for prenatal care).

**Table 1:** **Characteristics of pregnant women**^**a **^**and pregnant women**^**a **^**with a diagnostic code for CMV, MarketScan 2009**

**Characteristic**	**Pregnant women**^**a**^		**Pregnant women**^**a **^**with an ICD-9-CM diagnostic code for CMV disease**	
	**#**	**%**	**#**	**%**
**Age Group (years)**				
15-19	2,955	3.8%	0	0.0%
20-29	30,819	39.6%	4	33.3%
30-39	40,815	52.5%	8	66.7%
40-44	3,184	4.1%	0	0.0%
**Urban Residence**				
No	10,592	13.6%	2	16.7%
Yes	67,181	86.4%	10	83.3%
**Region**^**b**^				
Northeast	9,674	12.5%	1	8.3%
North Central	21,206	27.3%	3	25.0%
South	34,781	44.8%	5	41.7%
West	11,970	15.4%	3	25.0%
**Diagnostic Code for Infectious Mononucleosis**^**c**^				
No	77,729	99.9%	12	100.0%
Yes	44	0.1%	0	0.0%

Note: CMV, cytomegalovirus; ICD-9-CM, International Classification of Diseases-9^th^ revision, Clinical Modification.

^a^Pregnant women defined as female enrollees who were 1) aged 15–44 years, 2) enrolled for the entire year in 2009, and 3) had a delivery code (defined in Appendix) between October-December 2009.

^b^Excludes those pregnant women with other or unknown information on region.

^c^A diagnosis of mononucleosis was defined by an ICD-9-CM code for infectious mononucleosis (ICD-9-CM 075).

There were 1,668 (2%) pregnant women with a code for CMV-specific testing [Table 2], of whom 1,624 had a code for CMV IgG or IgM testing, 62 had a code for CMV PCR, 2 had a code for CMV DFA, and 2 had a code for CMV EIA. Among the 1,624 pregnant women with a code for CMV IgG or IgM testing, 72% had codes for both CMV IgG and IgM testing, 25% for CMV IgG testing alone, and 3% for CMV IgM testing alone. Among the 1,668 pregnant women with a code for CMV-specific testing conducted during pregnancy, half (52%) had testing performed in the 1^st^ trimester [Table 2]. Rates of CMV-specific testing were higher among older women, women living in the Northeast or an urban area, and women with a diagnostic code for mononucleosis [Table 3]. Only 44 (0.06%) pregnant women had a diagnostic code for mononucleosis; of these, 6 (14%) had a code for CMV-specific testing.

**Table 2:** **Type of CMV testing and timing of CMV testing among pregnant women**^**a **^**and among pregnant women**^**a **^**with a diagnostic code for CMV, MarketScan 2009**

	**Pregnant women**^**a **^**with CMV-specific testing**^**b**^		**Pregnant women**^**a **^**with an ICD-9-CM diagnostic code for CMV disease and CMV-specific testing**^**b**^	
	**N, 1,668**		**N, 10**	
	**#**	**%**	**#**	**%**
**Type of CMV-specific Test**^**b**^				
CMV PCR ONLY	17	1%	1	10%
CMV DFA ONLY	1	0%	0	0%
CMV Serology ONLY (CMV IgG, IgM, and EIA)	741	44%	5	50%
CMV PCR AND CMV Serology	8	0%	0	0%
CMV PCR AND Non-Specific Culture or PCR	24	1%	2	20%
CMV PCR, CMV Serology, AND Non-specific Culture or PCR	13	1%	0	0%
CMV DFA, CMV Serology, AND Non-Specific Culture or PCR	1	0%	0	0%
CMV Serology AND Non-specific Culture or PCR	863	52%	2	20%
**Gestational Age when CMV-Specific Testing Conducted**^**c**^				
1^st^ Trimester	863	52%	5	50%
2^nd^ Trimester	395	24%	4	40%
3^rd^ Trimester	410	24%	1	10%

Note: CMV, cytomegalovirus; ICD-9-CM, International Classification of Diseases-9^th^ revision, Clinical Modification; PCR, polymerase chain reaction; IgG, Immunoglobulin G; IgM, Immunoglobulin M; DFA, direct fluorescent testing; EIA, enzyme immunoassay.

^a^Pregnant women defined as female enrollees who were 1) aged 15–44 years, 2) enrolled for the entire year in 2009, and 3) had a delivery code (defined in Appendix) between October-December 2009.

^b^CMV-specific testing includes CMV IgG, CMV IgM, CMV DFA, CMV EIA, and CMV PCR testing.

^c^Date of first claim with a delivery code (defined in Appendix) was used to approximate delivery date in order to calculate the approximate age when the first CMV-specific testing was done. We assumed that CMV-specific testing which occurred 0–13 weeks was within the 1^st^ trimester, testing within weeks 14–26 to be in the 2^nd^ trimester, and testing within weeks 27–42 to be in the 3^rd^ trimester. Pregnant women with testing that occurred before (<0 weeks) or after (≥43 weeks) pregnancy were excluded.

**Table 3:** **Association between CMV-specific testing**^**a **^**by selected factors, pregnant women**^**b**^**, MarketScan 2009**

**Factor**	**Pregnant women**^**b**^			
	**Total**	**# Tested**	**% Tested**	**p-Value**
**Age Group (years)**				
15-19	2,955	22	0.7%	<0.001
20-29	30,819	519	1.7%	
30-39	40,815	1,022	2.5%	
40-44	3,184	105	3.3%	
**Urban Residence**				
No	10,592	65	0.6%	<0.001
Yes	67,181	1,603	2.4%	
**Region**^**c**^				
Northeast	9,674	628	6.5%	<0.001
North Central	21,206	313	1.5%	
South	34,781	579	1.7%	
West	11,970	148	1.2%	
**Diagnostic Code for Infectious Mononucleosis**^**d**^				
No	77,729	1,662	2.1%	<0.001
Yes	44	6	13.6%	

Note: CMV, cytomegalovirus; PCR, polymerase chain reaction; IgG, Immunoglobulin G; IgM, Immunoglobulin M; DFA, direct fluorescent testing; EIA, enzyme immunoassay; ICD-9-CM, International Classification of Diseases-9^th^ revision, Clinical Modification.

^a^CMV-specific testing includes CMV IgG, CMV IgM, CMV DFA, CMV EIA, and CMV PCR testing.

^b^Pregnant women defined as female enrollees who were 1) aged 15–44 years, 2) enrolled for the entire year in 2009, and 3) had a delivery code (defined in Appendix) between October-December 2009.

^c^Excludes those pregnant women with other or unknown information on region.

^d^A diagnosis of mononucleosis was defined by an ICD-9-CM code for infectious mononucleosis (ICD-9-CM 075).

To assess the sensitivity of the MarketScan database in capturing CMV-specific testing during pregnancy, we calculated frequencies of other routinely recommended prenatal laboratory tests that are conducted for all pregnant women as part of standard prenatal care [Table 4]. There were 71,002 (91%) pregnant women with ≥1 codes for other routinely recommended laboratory tests (i.e., rubella, anemia, urine, glucose, syphilis, hepatitis B, Chlamydia, and HIV); among these women with claims for these other laboratory tests, 1,660 (2.3%, range 1.5-4.2%) had CMV-specific testing [Table 4].

**Table 4:** **Rates of routinely recommended laboratory tests for pregnant women**^**a**^**, and rates of CMV-specific testing among pregnant women**^**a **^**with claims for routinely recommended laboratory tests, MarketScan, 2009**

**Routinely recommended laboratory tests for pregnant women**^**a**^	**Pregnant women**^**a**^	**Pregnant women**^**a **^**with CMV-specific testing**^**b**^
	**# (%)**	**# (%)**
Anemia testing	10,033 (13)	146 (1.5)
Glucose testing	54,989 (71)	1,228 (2.2)
Urine testing	54,947 (71)	1,311 (2.4)
Syphilis testing	65,047 (84)	1,613 (2.5)
Chlamydia testing	49,730 (64)	1,225 (2.5)
HIV testing	57,262 (74)	1,530 (2.7)
Hepatitis B testing	23,011 (30)	800 (3.5)
Rubella testing	15,546 (20)	650 (4.2)

Note: CMV, cytomegalovirus; PCR, polymerase chain reaction; IgG, Immunoglobulin G; IgM, Immunoglobulin M; DFA, direct fluorescent testing; EIA, enzyme immunoassay.

^a^Pregnant women defined as female enrollees who were 1) aged 15–44 years, 2) enrolled for the entire year in 2009, and 3) had a delivery code (defined in Appendix) between October-December 2009.

^b^CMV-specific testing includes CMV IgG, CMV IgM, CMV DFA, CMV EIA, and CMV PCR testing.

We identified 12 (0.02%) pregnant women with an ICD-9-CM code for CMV disease among the 77,773 pregnant women. Four (33%) were aged 20–29 years and 8 (67%) were aged 30–39 years [Table 1]. Ten (80%) had a code for CMV-specific testing performed during pregnancy, with half tested in the 1^st^ trimester [Table 2]. None of the pregnant women with an ICD-9-CM code for CMV had a diagnostic code for mononucleosis.

## Discussion

This is the first study to examine rates of prenatal CMV testing in the United States using national healthcare claims data. We found that the rate of claims for CMV testing among privately-insured pregnant women in 2009 was low (2%), which suggests that screening for CMV infection during pregnancy is not commonly performed. This finding is consistent with self-reported data from physicians in which only 1-2% reported routinely screening their pregnant patients for CMV infection [13,25]. Among those who reported ever testing for CMV during pregnancy, testing usually occurred in response to detection of a fetal abnormality or patient request for testing [13]. We did not expect to find evidence of widespread prenatal screening for CMV infection in the United States as there are no recommendations for it at this time from professional associations such as the American College of Obstetricians and Gynecologists [11,24]. In some European countries and in Israel, prenatal testing for CMV is more widely performed, even in the absence of recommendations for routine screening [26,27]. We found higher rates of testing among patients in our study population with a diagnosis of mononucleosis, a symptom potentially attributable to CMV infection, but the rate of testing for CMV was only 14% even in this high-risk group. Although CMV infection is often asymptomatic, CMV infection should be considered as part of the differential diagnosis in pregnant women who present with mononucleosis-like symptoms [13,22]. Understanding current obstetric CMV testing practices is important for identifying baseline testing practices, and, in the future, for monitoring implementation of screening and prevention programs as recommendations evolve.

Routine prenatal screening for CMV remains controversial and has not been endorsed by any professional organization or public health authority worldwide [11,12,27]. Data on the effectiveness of treatments for primary CMV infection in pregnancy are limited [4,20], although results from initial studies of treatment with CMV-specific hyperimmune globulin were promising [17]. In a study performed in Italy, they found that only 1 out of 31 pregnant women (3%) with primary CMV infection who received hyperimmune globulin gave birth to an infant with CMV disease, compared to 7 out of 14 pregnant women (50%) with primary CMV infection who did not receive hyperimmune globulin [17]. However, this was not a randomized controlled study and the efficacy of treatment with hyperimmune globulin could not be properly assessed. Data from randomized clinical trials in Europe and the United States could provide evidence needed for treatment of primary CMV infection with hyperimmune globulin [18-20]. Given the limited experience with CMV IgG avidity testing in clinical settings in the United States, algorithms for CMV counseling and screening, and effective treatment options, would need to be evaluated before routine prenatal screening for primary CMV infection could be recommended.

Another prenatal approach for prevention of congenital CMV is counseling of women to avoid exposures to CMV during pregnancy for all pregnant women, regardless of their CMV serostatus. The American College of Obstetricians and Gynecologists recommends counseling pregnant patients about thorough hand-washing when around young children to reduce CMV transmission [11]. Some data suggest that pregnant women are more likely to adopt and maintain practices to reduce household exposure to CMV if they are aware of their CMV seronegative status [28]. Routine determination of CMV serostatus before or early in pregnancy may enhance patient education efforts directed at reducing exposure to CMV during pregnancy to prevent maternal infection and subsequent vertical transmission. Data from a large prospective study in France reported that seroconversion rates in pregnant women told of their CMV seronegative status and counseled to adopt behavioral measures to reduce CMV exposure were 0.2%, significantly lower than estimated pre-intervention seroconversion rates in the study and expected rates for this population [28,29]. However, adherence to recommended preventative measures was not monitored and the study did not use a randomized design because the investigators deemed it unethical. Results of a survey of obstetricians and gynecologists in 2007 found fewer than half reported counseling their patients about preventing CMV infection [13]. Fuller implementation of recommendations for routine counseling and the addition of screening for CMV serostatus during pregnancy as part of risk reduction counseling in the United States would require provider education. In addition, careful consideration of the timing and reporting of such testing, as well as wider availability of CMV IgG avidity testing and standardization of commercial assays [10] would be required such that pregnant women identified as seropositive could be managed with additional diagnostic testing as appropriate.

There are a number of limitations to this study based on medical claims reported for insurance reimbursement purposes. We did not have access to laboratory results and it is not possible to link MarketScan claims data with medical records to validate our ascertainment of CMV testing. There is no CPT code for CMV IgG avidity testing and therefore we were unable to determine rates of IgG avidity testing. Claims data may underestimate laboratory tests that were performed if the provider failed to bill for the testing or it was not reimbursed by the enrollee’s insurance plan. Rates of CMV testing may be further underestimated in claims data if providers bill for CMV testing as part of a bundled claim for comprehensive prenatal care services under global billing rather than with a CMV-specific code. It seems unlikely however that CMV testing rates among pregnant women are much higher than those we report as we did not find substantially higher testing rates even among pregnant women for whom claims for other routine laboratory tests recommended during pregnancy were captured in the MarketScan Commercial database. It is unclear why rates of prenatal care for this privately insured population were lower than expected, especially for some routinely recommended tests such as anemia, hepatitis B, and rubella testing. Administrative claims data may not fully capture all prenatal care services, and this may be partially due to global billing. The MarketScan population is not representative of the national population since the data represents a large convenient sample primarily of individuals with private employer insurance, which accounts for 56% of the US population in 2009 [30]. People with employer-sponsored insurance are less likely to be low-income or non-white than are uninsured or publicly-insured people [31]. Separate MarketScan databases exist with healthcare claims data for the Medicaid population and it would be useful to examine CMV testing rates in the population with publicly-financed health insurance.

## Conclusions

This study serves as a baseline for understanding clinical awareness of and prenatal testing practices for CMV in the United States. Based on assignment of an ICD-9-CM code for CMV disease, we estimate the rate of CMV diagnosis during pregnancy in this population to be 0.02%. While 1-7% of susceptible pregnant women are estimated to develop CMV infection during pregnancy in the United States [6,29], there are limited data on the proportion of these infections that are symptomatic, lead to a medical visit, or result in diagnosis. Future studies may want to examine pregnant women with ultrasound results showing fetal abnormalities and the rates of maternal and fetal CMV testing among this group. The problem of maternal CMV infection during pregnancy and subsequent vertical transmission that results in neurologic impairments and hearing loss among children remains silent, despite its substantial public health burden [32]. There is currently no national surveillance for CMV infection or disease among pregnant women or infants and children. Healthcare claims and other administrative databases can be used to monitor uptake of medical services [33,34]. Use of these types of data are advantageous since the information is computerized and are available for large patient populations. As options for CMV prevention, diagnosis and treatment during pregnancy expand, there will be a growing need to monitor prenatal testing practices.

## Appendix

Appendix table 5 includes a list of ICD-9-CM (International classification of diseases, 9th revision, clinical modification) and CPT (Current procedural terminology) codes used for the study, including codes for CMV laboratory testing, pregnancy delivery, prenatal care, CMV diagnosis and CMV-related symptoms, and laboratory tests recommended as part of routine prenatal care.

**Table 5:** List of international classification of diseases, 9th revision, clinical modification (ICD-9-CM) and current procedural terminology (CPT) codes

**ICD-9-CM/CPT code(s)**	**Code description**
**CMV Laboratory Testing**	
CPT 86644-5	CMV Antibody Testing
CPT 87271	CMV Direct fluorescent antibody (DFA) Testing
CPT 87332	CMV enzyme immunoassay
CPT 87495-87497	CMV Infectious agent detection by nucleic acid (DNA or RNA)
CPT 87252, 87254	Non-specific virus isolation by culture
CPT 83890–1, 83898, 83900–2, 83904–9, 83912, 87800-1	Non-specific molecular diagnostics or infectious agent detection (DNA or RNA)
**Pregnancy Delivery**	
ICD-9-CM 650	Normal Delivery
ICD-9-CM 658.1-3	Premature rupture of membranes or delayed delivery after spontaneous, unspecified, or artificial rupture of membranes
ICD-9-CM 659	Other indications for care or intervention related to labor and delivery, not elsewhere classified
ICD-9-CM 66x.x	Complications of labor and delivery
ICD-9-CM 67x.x	Complications of Puerperium (period right after delivery)
ICD-9-CM V24.0	Postpartum care and examination, immediately after delivery
ICD-9-CM V27.x	Outcome of delivery
ICD-9-CM 69.02, 69.52	Dilation and curettage or aspiration curettage of uterus, following delivery or abortion
ICD-9-CM 72.x-74.x	Delivery procedures
CPT 01958, 01960–2, 01967-9	Anesthesia for delivery
CPT 59200	Insertion cervical dilator
CPT 59300	Episiotomy or vaginal repair
CPT 59400-59414	Vaginal Delivery
CPT 59510, 59514	Cesarean Delivery
CPT 59610, 59612, 59618, 59620	Delivery after previous cesarean delivery
**Prenatal Care**	
ICD-9-CM 64x.x	Complications of pregnancy
ICD-9-CM V22.xx	Normal pregnancy
ICD-9-CM V23.xx	Supervision high-risk pregnancy
ICD-9-CM V72.42	Pregnancy examination or test, positive result
ICD-9-CM 75.1	Diagnostic amniocentesis
ICD-9-CM 75.2	Intrauterine transfusion
ICD-9-CM 75.3	Other intrauterine operations on fetus and amnion
CPT 59000	Amniocentesis, diagnostic
CPT 59001-59076	Antepartum services
CPT 59618	Routine obstetric care including antepartum care, cesarean delivery, and postpartum care, following attempted vaginal delivery after previous cesarean delivery
CPT 59897	Unlisted fetal invasive procedure, including ultrasound guidance
CPT 76801-76828	Obstetrical ultrasound
CPT 76941	Ultrasound guidance for fetal transfusion or cordocentesis
CPT 76945	Ultrasound guidance for chorionic villus sampling
CPT 76946	Ultrasound guidance for amniocentesis
CPT 80055	Obstetric panel
CPT 82105-7	Serum alpha-fetoprotein
CPT 82143	Amniotic fluid scan (spectrophotometric)
CPT 82731	Fetal fibronectin
CPT 83030, 83033	Fetal hemoglobin
CPT 83632	Human placental lactogen
CPT 83661-4	Fetal lung maturity assessment
CPT 84163	Pregnancy-associated plasma protein-A
CPT 84702-3	Human chorionic gonadotropin
CPT 85460-1	Hemoglobin or rbcs, fetal, for fetomaternal hemorrhage
CPT 88235	Tissue culture of amniotic fluid or chorionic villus cells
CPT 88267	Chromosome analysis using amniotic fluid or chorionic villus cells
CPT 88269	In situ chromosome analysis for amniotic fluid cells
CPT 59400, 59425, 59426, 59510, 59610	Global billing code for routine prenatal obstetric care
**CMV Diagnosis and CMV-Related Symptoms**	
ICD-9-CM 078.5	CMV Disease
ICD-9-CM 075	Infectious Mononucleosis
**Routinely Recommended Laboratory Tests**	
CPT 82947–8, 82950–2, 83036-7	Glucose Test
CPT 81007, 81020, 87086, 87088, 87070–1, 87073, P7001	Urine Culture
CPT 86592–3, 80055, 86781	Syphilis Test
CPT 3513F, 80074, 86704, 86706, 87340, 87341	Hepatitis B Test
CPT 3511F, 86631, 86632, 87110, 87270, 87320, 87485, 87486, 87487, 87490, 87491, 87492, 87810; ICD-9 V739	Chlamydia Test
CPT 86689, 86701, 86702, 86703, 87390, 87391, 87534, 87535, 87536, 87537, 87538, 87539, G0432, G0433, G0435	HIV Test
CPT 85013–4, 85018	Anemia: Blood count with hematocrit, Blood count with hemoglobin

## Abbreviations

CMV: Cytomegalovirus; ICD-9-CM: International Classification of Diseases, 9^th^ revision, Clinical Modification; CPT: Current Procedural Terminology; PCR: Polymerase chain reaction; IgG: Immunoglobulin G; IgM: Immunoglobulin M; DFA: Direct fluorescent antibody; EIA: Enzyme immunoassay.

## Competing interests

The authors declare that they have no competing interests.

## Authors’ contributions

JL conceptualized and designed the study; acquired, analyzed, and interpreted the data; drafted and critically revised the manuscript for important intellectual content; and approved the final manuscript as submitted. MJC conceptualized and designed the study; interpreted the data; reviewed and critically revised the manuscript for important intellectual content; and approved the final manuscript as submitted. SDG conceptualized and designed the study; interpreted the data; reviewed and critically revised the manuscript for important intellectual content; and approved the final manuscript as submitted. SRB conceptualized and designed the study; interpreted the data; reviewed and critically revised the manuscript for important intellectual content; and approved the final manuscript as submitted. All authors read and approved the final manuscript.

## Pre-publication history

The pre-publication history for this paper can be accessed here:

http://www.biomedcentral.com/1471-2334/12/334/prepub
